# 6-Hydrazinylnicotinic acid: a powder study

**DOI:** 10.1107/S160053681200637X

**Published:** 2012-02-17

**Authors:** Mwaffak Rukiah, Atef Arfan

**Affiliations:** a Department of Chemistry, Atomic Energy Commission of Syria (AECS), PO Box 6091, Damascus, Syrian Arab Republic

## Abstract

The structure of the title compound, C_6_H_7_N_3_O_2_, is of inter­est with respect to radiopharmacueticals. The crystal packing is characterized by N—H⋯O and O—H⋯N hydrogen bonds, which form a three-dimensional network. The molecule is planar except for one of the amine H atoms.

## Related literature

For background on radiopharmacueticals, see: Callahan *et al.* (1996 ▶); Rennen *et al.* (2000 ▶). For general background, see: Abrams *et al.* (1990 ▶). For details of the synthesis, see: Schwartz *et al.* (1995 ▶). For geometric data, see: Allen *et al.* (1987 ▶). For descriptions of the powder diffraction profile, see: Thompson *et al.* (1987 ▶); Finger *et al.* (1994 ▶); Stephens (1999 ▶); Von Dreele (1997 ▶). For refinement by the LeBail method, see: Le Bail *et al.* (1988 ▶).
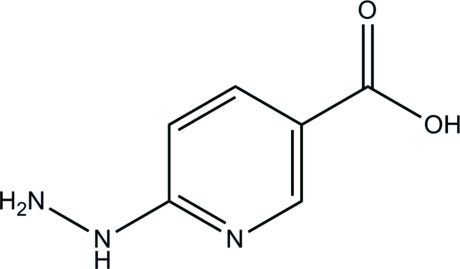


## Experimental

### 

#### Crystal data

C_6_H_7_N_3_O_2_
*M* *_r_* = 153.15
Monoclinic, 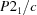

*a* = 6.69930 (14) Å
*b* = 13.8834 (2) Å
*c* = 7.10677 (9) Å
β = 91.7805 (11)°
*V* = 660.67 (2) Å^3^
*Z* = 4
Cu *K*α_1_ radiation
λ = 1.5406 Å
μ = 1.01 mm^−1^
*T* = 298 K
Flat sheet, 8 × 8 mm

#### Data collection

Stoe STADI P diffractometer
Specimen mounting: powder loaded between two Mylar foils
Data collection mode: transmission
Scan method: step
Absorption correction: for a cylinder mounted on the ϕ axis [flat-plate transmission absorption correction (*GSAS* absorption/surface roughness correction function number 4 with a non-refined term of μ*d* = 0.1482)] *T*_min_ = 0.732, *T*_max_ = 0.795
2θ_min_ = 9.969°, 2θ_max_ = 84.949°, 2θ_step_ = 0.02°

#### Refinement

*R*_p_ = 0.023
*R*_wp_ = 0.030
*R*_exp_ = 0.021
*R*(*F* ^2^) = 0.01796
χ^2^ = 2.016
4250 data points
146 parameters
26 restraints
Only H-atom coordinates refined

### 

Data collection: *WinXPOW* (Stoe & Cie, 1999 ▶); cell refinement: *FULLPROF* (Rodriguez-Carvajal, 2001 ▶) and *GSAS* (Larson & Von Dreele, 2004 ▶); data reduction: *WinXPOW*, *DICVOL04* (Boultif & Louër, 2004 ▶), and *CheckGroup* interfaced by *WinPLOTR* (Roisnel & Rodriguez-Carvajal, 2001 ▶); program(s) used to solve structure: *EXPO2009* (Altomare *et al.*, 2009 ▶); program(s) used to refine structure: *GSAS* interfaced by *EXPGUI* (Toby, 2001 ▶); molecular graphics: *ORTEP-3* (Farrugia, 1997 ▶); software used to prepare material for publication: *publCIF* (Westrip, 2010 ▶).

## Supplementary Material

Crystal structure: contains datablock(s) global, I. DOI: 10.1107/S160053681200637X/fy2040sup1.cif

Additional supplementary materials:  crystallographic information; 3D view; checkCIF report

## Figures and Tables

**Table 1:** Hydrogen-bond geometry (Å, °)

*D*—H⋯*A*	*D*—H	H⋯*A*	*D*⋯*A*	*D*—H⋯*A*
N2—H1N2⋯O1^i^	0.89 (3)	1.94 (2)	2.792 (8)	158 (4)
N3—H1N3⋯O2^ii^	0.87 (3)	2.39 (4)	2.967 (10)	124 (6)
N3—H2N3⋯O1^iii^	0.87 (3)	2.23 (6)	2.950 (11)	141 (5)
O2—H1O2⋯N1^iv^	0.822 (15)	1.818 (16)	2.622 (10)	165.2 (18)

Symmetry codes: (i) 
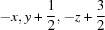
; (ii) 
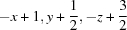
; (iii) 
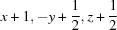
; (iv) 
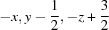
.

**Fig. 1.**
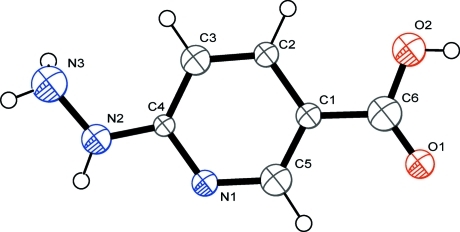
The molecule structure of (I), showing the atom numbering. Displacement ellipsoids are drown at the 50% probability level and H atoms are shown as small spheres of arbitrary radii.

**Fig. 2.**
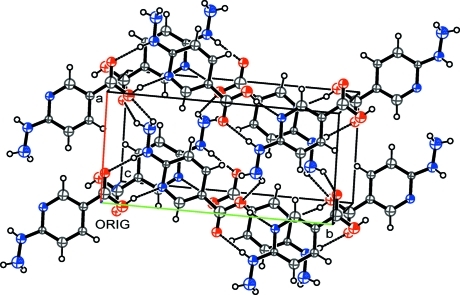
View of crystal packing of compound (I). Hydrogen bonds are shown as dashed lines.

**Fig. 3.**
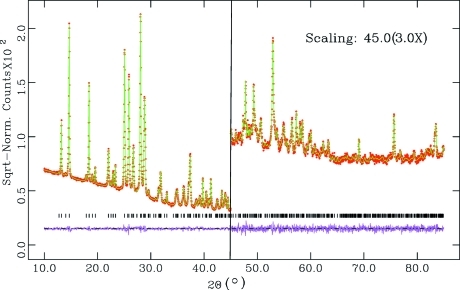
Final Rietveld plot of compound (I). Observed data points are indicated by dots, the best-fit profile (upper trace) and the difference pattern (lower trace) are solid lines. The vertical bars indicate the positions of Bragg peaks.

## supplementary crystallographic information

### Comment

6-Hydrazinylnicotinic acid (I) introduced by Abrams *et al.*, (1990), functions as a bifunctional chelating agent, forming a bridge between biomolecules and technetium (Callahan *et al.*, 1996; Rennen *et al.*, 2000). The (I) conjugated molecules react as monodentate ligands. Compound (I) is often used to synthesize bioconjugates for radiolabelling with ^99^*^m^*Tc and it is capable of efficient capture of technetium at extremely low concentrations. Compound (I) has a tendency to crystallize in the form of very fine pale yellow powder. Since no single-crystal of sufficient thickness and quality could be obtained, a structure determination by powder X-ray diffraction data was attempted. An *ORTEP* (Farrugia, 1997) view of compound (I) with atomic labeling is shown in Fig. 1. Bond lengths and angles in compound (I) are in their normal ranges (Allen *et al.*, 1987). The crystal packing is characterized by intermolecular hydrogen bonds involving the hydroxyl H atom and the amine H atom (Table 1).The hydrogen bonds form a three dimensional network (Fig. 2).

### Experimental

The synthesis of 6-hydrazinylnicotinic acid (I) was achieved according to the reported method (Schwartz *et al.*, 1995). 6-Chloronicotinic acid (8.0 g) was added to 35 ml of 85% hydrazine hydrate. The reaction mixture was heated at 373 K for 4 h. The homogeneous reaction mixture was concentrated to dryness to give a white solid. This solid was dissolved in water and on acidification to pH 5.5 with concentrated hydrochloric acid a precipitate formed. The precipitate was filtered and the solid was washed with 95% ethanol and ether to give 4.52 g of a pale brown solid (I); yield 58%.

^1^H and ^13^C{1H} NMR spectra were recorded in DMSO-D6 on a Bruker Biospin 400 spectrometer. IR spectrum was recorded on a Jasco FT–IR 300E instrument.

Spectroscopic data for (I): ^1^H NMR (DMSO-D6): δ 6.71 (*d*, 1H, py, J = 8.8 Hz),7.86 (*dd*, 1H, py, J = 2.4, 8.8 Hz), 8.52 (*d*, 1H, py, J = 2 Hz); ^13^C NMR (DMSO-D6) δ: 105.2 (py), 114.9 (py), 138.1 (py), 151.1 (py), 164 (py), 167.2 (CO); IR (KBr, ν, cm^-1^): 3309, 3231 (NH_2_).

### Refinement

For pattern indexing, the extraction of the peak positions was carried out with the program *WinPLOTR* (Roisnel & Rodriguez-Carvajal, 2001). Pattern indexing was performed with the program *DicVol4.0* (Boultif & Louër, 2004). The first 20 lines of powder pattern were completely indexed on the basis of monoclinic system. The absolute error on each observed line was fixed at 0.02° (2θ). The figures of merit are sufficiently high to support the obtained indexing results [*M*(20) = 40.2, F(20) = 62.6(0.0045, 71)]. The whole powder diffraction pattern from 10 to 95° (2θ) was subsequently refined with cell and resolution constraints (Le Bail *et al.*, 1988) with a space group without systematic extinctions in monoclinic system, *P*2/*m*, using the `profile matching' option of the program *FullProf* (Rodriguez-Carvajal, 2001). The best estimated space group in the monoclinic system was *P*2_1_/*c* which determined with the help of the program *Check Group* interfaced by *WinPLOTR* (Roisnel & Rodriguez-Carvajal, 2001). The number of molecules per unit cell was estimated to be equal to *Z* = 4, it can be concluded that the number of molecules in the asymmetric unit is *Z'* = 1 for the space group *P*2_1_/*c*.

The structure was solved *ab initio* by direct methods using the program *EXPO2009* (Altomare *et al.*, 2009). The model found by this program was introduced in the program *GSAS* (Larson & Von Dreele, 2004) implemented in *EXPGUI* (Toby, 2001) for Rietveld refinements. During the Rietveld refinements, the effect of the asymmetry of low-order peaks was corrected using a pseudo-Voigt description of the peak shape (Thompson *et al.*, 1987) which allows for angle-dependent asymmetry with axial divergence (Finger *et al.*, 1994). The two asymmetry parameters of this function *S/L* and *D/L* were both fixed at 0.0225 during the Rietveld refinement. An excluded region from 85 to 95° (2θ) was used, which leads to better molecular geometry.

Non-H atoms were not restrained, but several restraints on bonds lengths and angles were applied to H atoms (see below). A planar group restraints to the aromatic ring and the carboxyl group, including their H atoms were also applied.

The H atoms of the NH, NH_2_, OH groups were located in a difference map. The aromatic H atoms were positioned in their idealized geometries using a riding model with C—H = 0.99 Å. The coordinates of these H atoms restrained to the distances N—H = 0.89 (1) Å, N—H_2_ = 0.87 (1) Å, O—H = 0.82 (1) Å and C—H = 0.99 (1) Å. All H atoms were refined with isotropic displacement parameters (set to 1.2 times of the *U*_eq_ of the parent atom for aromatic H atoms and to 1.5 times of the *U*_eq_ of the parent atom for NH, NH_2_, OH groups).

Intensities were corrected from absorption effects with a µ*.d* value of 0.148. A spherical harmonics correction (Von Dreele, 1997) of intensities for preferred orientation was applied in the final refinement with 12 coefficients. The use of the preferred orientation correction leads to better molecular geometry with better agreement factors. The final Rietveld agreement factors are *R*_p_ = 0.023, *R*_wp_ = 0.030 *R*_exp_ = 0.022, χ^2^ = 1.904, and *R*_F_^2^ = 0.02438. The final Rietveld plot of the X-ray diffraction pattern is given in Fig. 3.

### Crystal data

**Table:**

C_6_H_7_N_3_O_2_	*F*(000) = 320
*M**_r_* = 153.15	*D*_x_ = 1.54 Mg m^−^^3^
Monoclinic, *P*2_1_/*c*	Cu *K*α_1_ radiation, λ = 1.5406 Å
Hall symbol: -P 2ybc	µ = 1.01 mm^−^^1^
*a* = 6.69930 (14) Å	*T* = 298 K
*b* = 13.8834 (2) Å	Particle morphology: Fine powder
*c* = 7.10677 (9) Å	pale brown
β = 91.7805 (11)°	flat sheet, 8 × 8 mm
*V* = 660.67 (2) Å^3^	Specimen preparation: Prepared at 298 K and 101.3 kPa
*Z* = 4	

### Data collection

**Table:**

Stoe STADI P diffractometer	Scan method: step
Radiation source: sealed X-ray tube	Absorption correction: for a cylinder mounted on the φ axis [Flat-plate transmission absorption correction (*GSAS* absorption/surface roughness correction function number 4 with a non-refined term of µd = 0.1482)]
Curved Ge(111) monochromator	*T*_min_ = 0.732, *T*_max_ = 0.795
Specimen mounting: powder loaded between two Mylar foils	2θ_min_ = 9.969°, 2θ_max_ = 84.949°, 2θ_step_ = 0.02°
Data collection mode: transmission	

### Refinement

**Table:**

Least-squares matrix: full	146 parameters
*R*_p_ = 0.023	26 restraints
*R*_wp_ = 0.030	Primary atom site location: structure-invariant direct methods
*R*_exp_ = 0.021	Secondary atom site location: difference Fourier map
*R*(*F*^2^) = 0.01796	Hydrogen site location: difference Fourier map
χ^2^ = 2.016	Only H-atom coordinates refined
4250 data points	(Δ/σ)_max_ = 0.03
Excluded region(s): The use of the excluded region from 85 to 95° (2θ) leads to better molecular geometry.	Background function: Shifted Chebyshev function of 1st kind (GSAS Background function number 1) with 15 terms 1: 1800.66 2: -1847.80 3: 941.880 4: -249.680 5: 12.6164 6: 58.5368 7: -22.4573 8: -39.9081 9: 32.2315 10: 0.665645 11: -20.8095 12: 16.0647 13: -6.68008 14: -5.95330 15: 5.95798
Profile function: GSAS CW profile function number 4 with 21 terms, i.e., pseudovoigt profile coefficients as parameterized in (Thompson *et al.*, 1987), asymmetry correction of Finger *et al.* (1994) and microstrain broadening by Stephens (1999). #1(GU) = 0.000 #2(GV) = 0.000 #3(GW) = 8.378 #4(GP) = 0.000 #5(LX) = 1.698 #6(ptec) = 0.00 #7(trns) = 0.00 #8(shft) = 0.0000 #9(sfec) = 0.00 #10(S/L) = 0.0225 #11(H/L) = 0.0228 #12(eta) = 0.6000 #13(S400 ) = 2.2E-01 #14(S040 ) = 3.7E-03 #15(S004 ) = 4.3E-01 #16(S220 ) = 3.8E-02 #17(S202 ) = 2.3E-01 #18(S022 ) = 5.1E-01 #19(S301 ) = -2.6E-01 #20(S103 ) = 1.6E-01 #21(S121 ) = -1.9E-01. Peak tails are ignored where the intensity is below 0.0010 times the peak. Aniso. broadening axis 0.0 0.0 1.0	Preferred orientation correction: spherical hamonics function

### Special details

**Table:**

Experimental. The sample was ground lightly in a mortar, loaded between two Mylar foils and fixed in the sample holder with a mask of 8.0 mm internal diameter.

### Fractional atomic coordinates and isotropic or equivalent isotropic displacement parameters (Å^2^)

**Table:**

	*x*	*y*	*z*	*U*_iso_*/*U*_eq_	
C1	0.0949 (15)	0.0911 (6)	0.7824 (15)	0.025 (3)*	
C2	0.3019 (11)	0.0838 (5)	0.8434 (12)	0.024 (3)*	
C3	0.4127 (11)	0.1653 (6)	0.8763 (12)	0.035 (4)*	
C4	0.3286 (12)	0.2568 (6)	0.8428 (14)	0.018 (3)*	
C5	0.0146 (12)	0.1829 (7)	0.7521 (12)	0.039 (5)*	
C6	−0.035 (2)	0.0069 (9)	0.7503 (17)	0.047 (4)*	
N1	0.1331 (11)	0.2628 (5)	0.7828 (11)	0.026 (3)*	
N2	0.4164 (12)	0.3424 (4)	0.8716 (12)	0.034 (3)*	
N3	0.6139 (10)	0.3455 (6)	0.9453 (13)	0.050 (3)*	
O1	−0.2097 (8)	0.0126 (4)	0.7110 (10)	0.032 (3)*	
O2	0.0600 (8)	−0.0758 (5)	0.7824 (11)	0.043 (3)*	
H2	0.362 (2)	0.0198 (11)	0.869 (3)	0.029 (4)*	
H3	0.554 (3)	0.1597 (10)	0.917 (3)	0.042 (4)*	
H5	−0.127 (3)	0.1905 (12)	0.707 (3)	0.047 (5)*	
H1N2	0.364 (4)	0.3948 (12)	0.816 (6)	0.052 (4)*	
H1N3	0.696 (2)	0.325 (5)	0.861 (5)	0.075 (5)*	
H2N3	0.644 (4)	0.4040 (17)	0.977 (10)	0.075 (5)*	
H1O2	−0.018 (3)	−0.1211 (10)	0.768 (4)	0.065 (4)*	

### Geometric parameters (Å, º)

**Table:**

C1—C2	1.444 (10)	C6—C1	1.471 (15)
C2—C3	1.369 (9)	C6—O1	1.198 (12)
C2—H2	0.990 (14)	C6—O2	1.328 (11)
C3—C4	1.407 (10)	N2—C4	1.339 (7)
C3—H3	0.981 (14)	N2—N3	1.409 (7)
C4—N1	1.367 (9)	N2—H1N2	0.89 (3)
N1—C5	1.378 (9)	N3—H1N3	0.87 (3)
C5—C1	1.397 (12)	N3—H2N3	0.87 (3)
C5—H5	0.997 (14)	O2—H1O2	0.824 (14)
			
C2—C1—C5	118.2 (7)	C1—C5—H5	120.3 (13)
C2—C1—C6	123.3 (9)	N1—C5—H5	120.2 (13)
C5—C1—C6	118.6 (9)	C1—C6—O1	123.5 (12)
C1—C2—C3	120.3 (6)	C1—C6—O2	112.5 (10)
C1—C2—H2	119.9 (6)	O1—C6—O2	123.8 (13)
C3—C2—H2	119.7 (6)	C4—N1—C5	122.8 (8)
C2—C3—C4	120.3 (7)	C4—N2—N3	119.2 (7)
C2—C3—H3	119.8 (6)	C4—N2—H1N2	119 (2)
C4—C3—H3	119.8 (6)	N3—N2—H1N2	119.4 (18)
C3—C4—N1	118.9 (7)	N2—N3—H1N3	110 (2)
C3—C4—N2	127.1 (8)	N2—N3—H2N3	110 (2)
N1—C4—N2	113.9 (8)	H1N3—N3—H2N3	110 (5)
C1—C5—N1	119.5 (6)	C6—O2—H1O2	109.9 (15)
			
C4—N1—C5—C1	−0.1 (13)	C5—C1—C2—C3	2.2 (14)
C5—N1—C4—N2	−177.5 (8)	C2—C1—C6—O2	−0.5 (15)
C5—N1—C4—C3	−0.5 (13)	C5—C1—C6—O1	−4.9 (17)
N3—N2—C4—N1	175.9 (8)	C2—C1—C6—O1	175.2 (10)
N3—N2—C4—C3	−0.8 (15)	C5—C1—C6—O2	179.4 (9)
C2—C1—C5—N1	−0.7 (13)	C1—C2—C3—C4	−2.9 (13)
C6—C1—C5—N1	179.5 (9)	C2—C3—C4—N2	178.6 (9)
C6—C1—C2—C3	−178.0 (10)	C2—C3—C4—N1	2.1 (13)

### Hydrogen-bond geometry (Å, º)

**Table:**

*D*—H···*A*	*D*—H	H···*A*	*D*···*A*	*D*—H···*A*
N2—H1*N*2···O1^i^	0.89 (3)	1.94 (2)	2.792 (8)	158 (4)
N3—H1*N*3···O2^ii^	0.87 (3)	2.39 (4)	2.967 (10)	124 (6)
N3—H2*N*3···O1^iii^	0.87 (3)	2.23 (6)	2.950 (11)	141 (5)
O2—H1*O*2···N1^iv^	0.822 (15)	1.818 (16)	2.622 (10)	165.2 (18)

Symmetry codes: (i) −*x*, *y*+1/2, −*z*+3/2; (ii) −*x*+1, *y*+1/2, −*z*+3/2; (iii) *x*+1, −*y*+1/2, *z*+1/2; (iv) −*x*, *y*−1/2, −*z*+3/2.
